# Impacts on Children and Adolescents’ Lifestyle, Social Support and Their Association with Negative Impacts of the COVID-19 Pandemic

**DOI:** 10.3390/ijerph18094780

**Published:** 2021-04-29

**Authors:** Shimin Zhu, Yanqiong Zhuang, Patrick Ip

**Affiliations:** 1 Department of Applied Social Sciences, The Hong Kong Polytechnic University, 11 Yuk Choi Road, Hung Hom, Kowloon, Hong Kong, China; yanqiong.zhuang@polyu.edu.hk; 2 Department of Paediatrics and Adolescent Medicine, LKS Faculty of Medicine, The University of Hong Kong, Pokfulam, Hong Kong, China; patricip@hku.hk

**Keywords:** school closure, COVID-19, children and adolescents, lifestyle, social support, mental health

## Abstract

The impacts of the COVID-19 pandemic on physical and mental health-related behaviors among children and adolescents are likely to be profound and long-lasting. This study aimed to investigate the changes in lifestyle and social support and their associations with negative impacts due to the pandemic. A classroom survey using stratified random sampling and structured questionnaire was conducted among Hong Kong primary and secondary school students. The paper-and-pen survey, administered by well-trained research assistants, was completed by 2863 participants aged 9–17 years old (M = 12.6, SD = 1.3) at a brief school reopening six months after the outbreak of the COVID-19 pandemic. About 48% and 37% of the participants stated that they paid increased attention to physical and mental health, respectively. About 20% to 40% stated that they found more support from their friends and family members; only a small percentage reported decreased social support. Around 25% to 50% spent more time to rest, relax, and exercise. The aforementioned changes varied among genders, education groups, and socio-economic status. In general, higher perceived vulnerability, feeling more stressed, apprehensive, and helpless were associated with more reported positive lifestyle changes, including more social/family support, increased mental health awareness, and a positive lifestyle. These positive changes serve as important cushions against the negative impacts of COVID-19.

## 1. Introduction

The Coronavirus disease (COVID-19) pandemic is having a profound and long-term impact on children and adolescents. Around 144 countries closed schools to contain the transmission of the virus, and more than 1.2 billion students around the world were “out of the classroom” due to the social distancing measure [1]. The lockdown intervention succeeded in reducing the exponential transmission of COVID-19 and achieved its intended positive health outcomes. However, such major lockdowns have also led to other changes in life. Many studies have highlighted the negative impact on physical and mental health [2,3,4,5], such as more screen time and sedentary behavior [6,7], more emotional distress, feeling anxiety, fear, frustrations, and loneliness [8]. Like other infectious diseases and macro-level crises, such as malaria, plague, cholera, HIV/AIDS, and SARS, COVID-19 is a threat to life [9], but it may also be a contributor to change lifestyles and social life. Recent studies found some positive lifestyle changes among adults, for example, improvement in healthy eating behavior in India and Italy [6,10], more physical activity, and increased quality time with family in Scotland [11]. These positive changes in lifestyle and social support may help people cope with the negative impacts. However, the data about positive changes are emerging and yet scarce, especially among children and adolescents. Thus, this study aims to examine children and adolescents’ changes in lifestyle and social support during COVID-19 in Hong Kong.

Children and adolescents are more vulnerable to mental distress during the pandemic [12]. Although COVID-19 is less threatening to children and adolescents in terms of infectious morbidity and mortality [13], the lockdown and quarantine measures have brought new challenges and negative impact on their mental health. For example, anxiety, depression, and post-traumatic symptoms were observed in children and adolescents [14]. A recent study among parents in Hong Kong reported increased children’s psychosocial problems and fewer prosocial behaviors [15]. Furthermore, some negative lifestyle changes were documented, such as increased screen time, sugar intake, and sedentary time [16,17].

On the other hand, the COVID-19 pandemic may also increase the awareness of the importance of health, thereby improving healthy lifestyle, such as increased time to sleep and relaxation, as well as healthier eating [18]. In Hong Kong, some positive lifestyle changes among adults were documented during the Severe Acute Respiratory Syndrome (SARS) in 2003 [19]. People were more aware of the importance of physical and mental health and spent more time for rest, relaxation, and social support [19]. Feeling vulnerable is also likely to be associated with more resilience and adaptation to coping with the uncontrollable changes in environment [20]. For example, feeling horrified and worried about the life-threatening disease may increase adults’ awareness of the importance of physical and mental health; feelings of helplessness may increase reaching out for help from friends and families [19]. Feeling stress is likely to increase time for rest and relaxation [19]. Increasing social support is found to be effective in helping children and adolescents to cope with the stress raised by the crisis [21,22,23,24]. Importantly, as children adolescence is the key stage of developing social function and establishing physical and mental health lifestyles, the pandemic’s impact on children and adolescents’ lifestyle and social life will be likely to continue in the near term and may also last across their lifespan [25,26]. Thus, it is important to examine the changes of children and adolescents’ lifestyle and social life during the pandemic and their association with a perceived negative impact.

The changes in lifestyle and social support may vary among gender, age, and socio-economic resources. As reported in the surveys in Scotland and the United States, younger, married, employed adult females were more likely to report a positive change in life [11,27]. Inner attributes such as gender and age, and social-ecological factors such as socio-economic factors, perceived safety at home and community are related to one’s reaction to risk [20]. Children in primary school and adolescents in secondary school may respond to the challenge of COVID-19 differently due to the educational approach and school life setting. To our knowledge, there is a lack of evidence that evaluates the specific changes in lifestyle and social life of different groups of children and adolescents.

This study investigated the potential changes in children and adolescents’ lifestyle, social support, and mental health due to the COVID-19 pandemic and their differences in gender, school types, and socio-economic status. The COVID-19 has been spreading rapidly since late 2019, and by March 2020, almost one-third of the world was under lockdown in order to tackle the COVID-19 pandemic [28]. In Hong Kong, the coronavirus started to spread in late January 2020 and the government announced city-wide school closures to prevent the spread of the virus. As one of the key strategies adopted to confine the spread of COVID-19 in Hong Kong, all schools including universities, secondary and primary schools, and kindergartens were ordered to close at the end of January 2020 by the government [29]. During this period of time, all libraries, playgrounds, beaches, and other sports facilities were closed as mandatory measures [30]. The government encouraged home office and advocated that citizens should stay at home and avoid outdoor activities as much as possible, but adults and children were still allowed to go outdoors without legal restrictions. Hong Kong was one of the first cities to mandate school closures during the COVID-19 outbreak when students did not go to school and studied at home for months until the epidemic was slightly improved in May. Schools were reopened on 27 May 2020 until the summer holiday started in early July. It was during this month that the survey was conducted to collect information about the impacts of COVID-19 on children and adolescents. We hypothesized that improved lifestyle changes and social support among children and adolescents were positively associated with their perceived negative impacts of the COVID-19 pandemic.

## 2. Materials and Methods

### 2.1. Procedure and Participants

Primary and secondary school students were invited to participate in the questionnaire survey. All study materials and procedures were approved by the Human Subjects Ethics Sub-Committee of the Hong Kong Polytechnic University (No. HSEARS20161222006) before the recruitment of schools. Informed consent was obtained from the parent of each participating student. We aimed to have a sample that could yield a margin of error of 0.98/(*n*) ^ 0.5 = 1.8%. Altogether, 4635 students and their parents from 4 primary schools and 13 secondary schools were approached, and 2863 students filled out the survey. We stopped the recruitment after the targeted sample size was reached. The response rate was 61.8%. The survey was anonymous, and all participants who finished the survey in classes with the presence of research assistants received souvenirs worth HKD$40 (US$5). Data entry was done by trained undergraduate student assistants of the university and double-checked by a senior research assistant.

A total of 2863 fourth to eighth grade students (aged 9 to 17) filled out the structured questionnaire. Five hundred ninety-nine (20.9%) primary school students and 2264 (79.1%) secondary school students (Grade 4: *n* = 181, 6.3%; Grade 5: *n* = 208, 7.3%; Grade 6: *n* = 210, 7.3%; the mean age of primary school participants = 10.8, SD = 1.0; Grade 7: *n* = 1209, 42.2%; and Grade 8: *n* = 1055, 36.8%; the mean age of secondary school participants = 13.1, SD = 0.9) completed the questionnaires.

### 2.2. Measures

#### 2.2.1. Impacts on Lifestyle Changes and Social Support

The mental health lifestyle scale [19] was used to measure the positive impacts of COVID-19 on lifestyle changes. The scale was adapted from the items which were used to measure people’s lifestyle changes during the Severe Acute Respiratory Syndrome (SARS) in Hong Kong in 2003 [19]. The adapted scale consisted of 5 items. Each item asked about the participants’ perceived changes in their lifestyle during the pandemic as compared to the pre-COVID19 period. Two items measured whether respondents were paying more or less attention to their physical and mental health conditions. Three items were used to measure whether the participants spend more or less time to rest, engage in relaxation activity, and exercise, respectively. Three choices were provided (increased, same as before, and decreased) and participants chose one option that best described them. These items measure subjective overall perceived changes.

Impacts on social support were measured with an instrument adapted from the one that measured the perceived changes in social support from family and friends during the SARS epidemic, compared to their situation before the epidemic [19]. The scale consisted of 5 items, including whether getting increased or decreased support from friends, getting increased or decreased support from family members, having more or less sharing of feelings with other family members, having more or less sharing of feelings with others when feeling blue, and whether becoming more caring for family members’ feelings (using a 3-point Likert scale: 1 “decreased”, 2 “same as before”, 3 “increased”). Cronbach’s alpha of the five items of social support was 0.76.

#### 2.2.2. Negative Impacts on Emotional States and Perceived Vulnerability

Negative impacts on mental health were measured with five dichotomous questions [19] asking whether respondents felt more stressed, more academic stress, more horrified, more apprehensive, and felt more helpless during the outbreak of the COVID-19 pandemic, compared to the period before with a Yes/No answer. Cronbach’s alpha was 0.80.

Perceived vulnerability to the coronavirus disease was measured with the adapted Perceived Risk of the HIV scale [31]. Six items measure how often the participant felt worry about themselves and their community being infected with the coronavirus disease using a 5-point Likert scale ranging from 1 “never” to 5 “always”. Sample items were I feel vulnerable to COVID-19 and I am worried that my local community would have new cases of COVID-19 infection. Items were averaged to create a mean score ranging from 1 to 5, higher scores indicating higher perceived vulnerability. The Cronbach’s alpha was 0.84.

The items I feel safe living in my community and I feel safe at home, using a 5-point Likert scale ranging from 1 “strongly disagree” to 5 “strongly agree”, were used to assess the sense of security under the COVID-19 pandemic. A higher score indicated feeling more safety in the community and at home during the pandemic.

#### 2.2.3. Sociodemographic and Socioeconomic Information

Family Affluence Scale (FAS) [32] was adapted and used in this study to measure children and adolescents’ family affluence levels. Participants were asked four items that reflected child’s family expenditure and consumption that were relevant to family affluence. The FAS was simple to answer, non-intrusive and non-sensitive; comprised multiple component items; and was relevant to contemporary economic circumstances [33,34]. We adapted the FAS into a four-item measure: (1) Does your family own a private car? (0: No; 1: Yes); (2) Do you have these learning devices: desktop computer, notebook computer, iPad or tablets, smartphone, and/or e-book reader? Select all that apply. (1: 1–2 pieces; 2: 3–5 pieces); (3) Does your home have these household appliances: television, refrigerator, microwave, washing machine, dishwasher, audio-visual equipment, air-conditioner? Select all that apply. (1: 1–3 pieces; 2: 4–7 pieces); (4) Do you have a satisfactory internet connection at home for your needs of daily life and learning? (0: very unsatisfactory—unsatisfactory; 1: satisfactory—very satisfactory). Items were combined to create a sum score ranging from 2 to 6; higher scores indicated more affluence. In addition, endorsing all lower options (total score = 2) was identified as low family affluence, while endorsing all upper options (total score = 6) was identified as high family affluence. Scoring 3–5 in FAS was identified as medium family affluence.

Gender, age, grade, and family structure were measured as demographic information. Family structure was measured with whether the respondents lived with both parents, with a single parent, or with neither parent. Parental educational level and parental occupation were asked.

### 2.3. Analysis

Data were analyzed using SPSS 22.0 (IBM, New York, Armonk, USA). The frequency and percentage were used to describe the demographic characteristics of participants. Chi-square test and *t*-test were used to examine the differences in impacts of COVID-19 between male and female students, primary and secondary school students, and low and high family affluence groups.

Logistic regression was conducted to examine how the increased negative emotion during the pandemic contributed to the changes in lifestyle and social support. The association (Odds Ratio), adjusting for age, gender, family affluence, was calculated in binary logistic regression analysis.

## 3. Results

### 3.1. Socio-Demographic Characteristics

Participant characteristics were summarized in Table 1. The age range of participants was 9–17 years (mean = 12.6, SD = 1.3). Males comprised 1346 (47.0%) of participants. Majority of students lived with both parents (*n* = 2282, 79.9%), 324 (11.3%) lived with their mother, 109 (3.8%) lived with their father, and 103 (3.6%) did not live with a parent. Seventy-seven (2.7%) students were identified as in the low family affluence group, 73.2% in the medium group, and 11.0% in the high group. Most of the participants did not know their parent’s educational level and reported that their father (66.5%) and mother (49.9%) had a paid job.

### 3.2. The Positive Changes in Lifestyle and Negative Impacts

Noticeable proportions of self-perceived positive changes in lifestyle during the COVID-19 pandemic were reported. Participants paid more attention to their physical health (48.4%), mental health (36.9%), and spent more time resting (48.2%) and relaxing (52.7%). However, more participants reported decreased time to exercise (33.3%) than those who reported increased time to exercise (25.0%). Female students were more likely than male students to pay attention to physical health (*χ*^2^ = 26.24, *p* < 0.001) and mental health (*χ*^2^ = 10.73, *p* < 0.0) and to spend more time to relax (*χ*^2^ = 11.64, *p* < 0.001). More primary than secondary school students increased their attention to physical health (*χ*^2^ = 8.38, *p* < 0.05), and more primary than secondary school students decreased time spent resting (*χ*^2^ = 11.56, *p* < 0.01) and relaxing (*χ*^2^ = 32.03, *p* < 0.001). Participants with high family affluence reported more positive changes in lifestyle in all items than those from low family affluence (see Table 2).

### 3.3. Positive Changes in Social Support

Table 3 presents the proportion of social support changes. More than half of participants reported that they perceived the same social support as before (57.0–67.9%). The proportion reporting increased social support (19.8–37.2%) was more than those reporting decreased social support (5.8–14.9%). More girls than boys shared feelings with others when feeling blue (21.9% vs. 17.5%) and cared for family members’ feelings (38.9% vs. 35.5%). On the contrary, more boys than girls reported decreased support from friends (12.3% vs. 9.1%) and decreased support from family members (8.1% vs. 6.4%). Compared to participants, more primary school participants reported increased support from friends (24.9% vs. 20.5%) and family (39.2% vs. 24.9%). Moreover, more primary school participants reported decreased support from friends (18.4% vs. 8.6%) and family (11.1% vs. 6.2%). The proportions of secondary school students who felt the same social support were significantly more than those among primary school students, (*χ^2^* = 44.21–76.96, all *p*s < 0.001). Participants from the low family affluence group reported greater decrease of social support and less increased social support than those from high socio-economic families (*χ^2^* = 13.82–34.31, all *p*s < 0.001).

### 3.4. Negative Mental Health Changes Due to COVID-19

The negative impact on mental health is presented in Table 4 and Table 5. Furthermore, 21.7–39.1% of participants felt more negative emotion and 58.4% of participants felt more study pressure during the COVID-19 pandemic. Female students were more likely than male students to feel more stress (*χ*^2^ = 29.95, *p* < 0.001), more study pressure (*χ^2^* = 83.31, *p* < 0.001), more horrified (*χ*^2^ = 22.05, *p* < 0.001), more apprehensive (*χ*^2^ = 36.40, *p* < 0.001), and more helpless (*χ*^2^ = 21.23, *p* < 0.001). Similarly, female students were more vulnerable than male students when considering infection of COVID-19 (see Table 4, *T* = −2.10, *p* < 0.05). Secondary school participants reported more study stress and feeling more apprehensive. Significantly more participants from the low family affluence group reported feeling horrified about COVID-19 and lower perceived safety at home and in their community (see Table 5).

### 3.5. Associations between Positive Impact and Negative Impacts

The associations between items representing increasing positive and negative impacts due to COVID-19 are summarized in Table 6 and Table 7. Those who perceived more vulnerability were more likely than others to have experienced the increasing positive lifestyle changes. The odds ratios were statistically significant for all item indicators of positive lifestyle changes (significant adjusted ORs ranged from 1.20 to 1.93). Perceiving safety at home was positively associated with the increasing concerns for a positive lifestyle and social support changes except spending more time to exercise (significant adjusted ORs = 1.18–1.37).

Feeling more stressed was significantly associated with spending more time on relaxation (OR = 1.40, 95% CI 1.12, 1.75) and sharing feelings with family (OR = 1.39, 95% CI 1.08, 1.78). Feeling more horrified was significantly associated with the increase of concern for mental health (OR = 1.50, 95% CI 1.13, 1.98). Moreover, feeling more apprehensive was significantly associated with the increase of concern for physical health (OR = 1.38, 95% CI 1.07, 1.79) and cared family’s feeling (OR = 1.30, 95% CI 1.01, 1.68). Feeling more helpless was significantly associated with more support from friends (OR = 1.65, 95% CI 1.20, 2.26) and more sharing of feelings with others when feeling blue (OR = 1.48, 95% CI 1.06, 2.06), but negatively associated with concern for physical health (OR = 0.71, 95% CI 0.54, 0.94).

Furthermore, the family affluence was positively associated with positive lifestyle changes and social support (OR 1.11–1.34, *p*s < 0.05). Students with more family affluence were more likely to have had positive lifestyle changes and perceived more social support.

## 4. Discussion

Despite concerns about the negative impact of COVID-19 on children and adolescents, there has been little research on their coping and adaptation in physical and mental health-related lifestyle. As children and adolescents are in a fast-developing period, the health-related lifestyle changes and negative impacts due the pandemic can have a profound impact on public health. The current study provides the first empirical evidence that positive changes in children and adolescents’ lifestyle and social support due to the pandemic have been fostered in midst of the negative impacts. Repeatedly experiencing school closure and irregular daily routines during the pandemic, a proportion of respondents reported feeling more vulnerable, helpless, horrified, apprehensive, and more stressed. However, a noticeable proportion of respondents exhibited better mental health awareness, healthier lifestyle, and better family and social support. Participants who reported higher perceived vulnerability also reported the increase of social supports. Children and adolescents may seek more positive lifestyle changes and positive social support to cope with the psychological distress caused by the pandemic [35]. Although in many cases, more than half of the respondents reported no changes, the percentages reporting favorable changes were much larger than the percentage reporting unfavorable changes. The sudden occurrence of a dramatic crisis may have made people reconsider priorities in their lives [19]. Family members were more likely to be spending time together during quarantine and working-from-home policy [36,37]. The better physical and mental health awareness and social support were in line with the local and worldwide intensive media coverage promoting precautionary measures and harmonious atmosphere [38]. The only aspect of unfavorable change documented in the current study was that more respondents reported decreasing the time spent on exercise than those reporting increased time for exercise, which is consistent with the findings among the general population worldwide [39,40,41].

Although it is not certain whether such observed positive changes would last, the magnitude of the reported changes was very noticeable. In the short term, positive lifestyle and social life changes, such as increasing time for physical and mental health caring and more time to rest, may be a buffer for children adolescents to secure more safety and control [19]. In the long run, these changes may have long-term impacts on child development in lifestyle and quality of life. Longitudinal studies are warranted for further examination.

Our study reported group differences among gender, school education, and socioeconomic status (SES) in both negative impacts and positive changes. Generally, female, primary school students, and students in higher SES family were found more likely to have favorable adaptation in physical and mental health lifestyles. Although more female participants reported negative emotions, such as feeling more stressed, apprehensive, and horrified, they were also more likely to pay increased attention to physical and mental health, spend more time to relax, and share feelings with family and friends. On the contrary, more male participants reported decreased social support during school closure in the pandemic. Females were more likely to engage in social support as coping strategies [11,42].

Primary and secondary school students did not differ much in negative impacts except that secondary school students reported more study stress and feeling more apprehensive. Generally, more primary school students paid increased attention to physical health and spent more time exercising, while more secondary school students spent increased time to rest and for relaxation. In terms of social support, the responses of primary school students were more polarised. Relatively large proportions of primary school participants reported both increased and decreased social supports, while more secondary school students reported no change in social support. Social media and instant message are popular tools for social support, and secondary school students may be more adapted to use online interaction for virtual support from peers during the pandemic. Primary school students may be more vulnerable and rely more on families for social support.

Family factors also matter. Respondents in the higher and lower end of SES exhibited a few significant differences in negative impact on mental health, which was generally consistent with large local and mainland large-scale local surveys [15,43]. More respondents in lower SES reported feeling helpless and horrified, while more higher-SES students reported increased study stress. There were also significant differences in favorable lifestyle changes and social supports. More participants in lower SES reported decreased social support and unfavorable lifestyle changes, while those in high SES reported more favorable lifestyle changes and enhanced social support. COVID-19 has intensified the financial pressure for low SES families and increased job insecurity. Parents may need to work longer to secure a job and maintain the daily necessities for the family [44]. The holistic quality of life of students in low-income families is more likely to be threatened [45] and their risk of psychosocial problems and problem behaviors is higher [15,46]. For example, inadequate digital devices and unsatisfactory internet access may limit their social connection with peers and teachers, limit the access to health-related information and knowledge, and increase risks of exposure to gaming temptation during lockdown [46]. Families trapped in smaller apartment may have greater difficulties of maintaining life quality [45] especially for low SES families. The current study also suggested that perceived safety at home was positively associated with most of the favorable changes. Students had more time to stay at home during city lockdown. Prolonged stay at home would create more quality parent-child interaction; it may also create more conflict, but time at home is more likely to foster favorable changes if home is a safe haven. The results of the study indicated the urgency as well as the importance of offering more supports to children and adolescents and their families to buffer the challenges they faced during and after the pandemic.

## 5. Limitations

This is a school-based classroom survey with sufficient instructions and ensured its anonymous nature. The response rate was high and students would love to share their lives after long term lockdown. The large magnitude of self-reported changes among a relatively large sample lends confidence that the reported changes were genuine. However, the present study had several limitations. This study was a cross-sectional one, which could not provide causal relationships and could not inform whether the positive changes may last. Further longitudinal studies are needed to study the long-term impact of the COVID-19 pandemic on child development of this generation and to examine the causal relationship of factors.

Furthermore, most of the measures were self-reported and used among adults in previous studies. We conducted a pilot study to make sure children could understand the questions, and well-trained research assistants in classroom provided explanation if needed. Future studies can develop more child and adolescent validated scales and objective parent or teacher reported measures to further test the impacts of the pandemic. Furthermore, the lifestyle changes included in this study were limited. Future studies should include sleep duration and sleep pattern, such as delayed bedtime. The impacts of COVID-19 on children and adolescents’ living experiences largely depended on the local epidemic situation, the prevention policy, the media orientation, and so on. Thus, the findings in this study should be taken into more consideration when generalized to the different populations outside Hong Kong.

## 6. Conclusions

The study focused on both the positive and negative impacts on children and adolescents’ lifestyle, mental health, and social support during school closure in the COVID-19 pandemic. Among the participants, female, primary school students, and students in higher SES families were found to be more likely to have favorable adaptation in physical and mental health lifestyles. Despite of the negative impacts of the pandemic on mental health, noticeable positive changes were also captured. Such positive effects may have served as a cushion against the negative impacts due to COVID-19. This original study has contributed to the scant literature on the study of children and adolescents’ lifestyle and social support during the pandemic.

## Figures and Tables

**Table 1:** Demographic characteristics (*N* = 2863).

		*N* (%)
School		
	Primary school	599 (20.9%)
	Secondary school	2264 (79.1%)
Gender		
	Male	1346 (47.0%)
	Female	1502 (52.5%)
	Missing	12 (0.5%)
Live with parent		
	With both parents	2282 (79.7%)
	With Dad	109 (3.8%)
	With Mom	324 (11.3%)
	With neither parent	103 (3.6%)
	Missing	45 (1.6%)
Family affluence		
	Low group	77 (2.7%)
	Medium group	2096 (73.2%)
	High group	315 (11.0%)
	Missing	375 (13.1%)
FAS (1)	Family Car ownership	
	No	1848 (64.5%)
	Yes	693 (24.2%)
	Do not know	322 (11.2%)
FAS (2)	Learning devices	
	1–2 pieces	1436 (50.2%)
	3–5 pieces	1400 (48.9%)
	Missing	27 (0.9%)
FAS (3)	Household appliances	
	1–3 pieces	392 (13.7%)
	4–7 pieces	2420 (84.5%)
	Missing	51 (1.8%)
FAS (4)	Internet	
	Unsatisfying	556 (19.4%)
	Satisfying	2263 (79.0%)
	Missing	44 (1.5%)
Parent education		
	Primary school (Dad/Mom)	112 (3.9%)/122 (4.3%)
	Secondary school (Dad/Mom)	1034 (36.1%)/1075 (37.5%)
	University or above (Dad/Mom)	577 (20.2%)/569 (19.9%)
	Do not know (Dad/Mom)	1140 (39.8%)/1097 (38.3)
Parent occupation		
	Dad/Mom has a paid job	1905 (66.5%)/1429 (49.9%)
	Dad/Mom is sick	20 (0.7%)/9 (0.3%)
	Dad/Mom is retired	44 (1.5%)/7 (0.2%)
	Dad/Mom is looking for a job	33 (1.2%)/37 (1.3%)
	Dad/Mom takes care of family	35 (1.2%)/606 (21.2%)
	Do not know (Dad/Mom)	826 (28.9%)/775 (27.1%)

FAS: Family Affluence Scale. Learning devices pieces: desktop computer, notebook computer, iPad or tables, smartphone, e-book reader. Household appliances pieces: television, refrigerator, microwave, washing machine, dishwasher, audio-visual equipment, air-conditioner.

**Table 2:** The positive impact on lifestyle changes by groups with Chi-Square (χ^2^) Test (*N* = 2863).

%	Gender			Primary or Secondary School			Family Affluence Group			Overall
	Male	Female	χ^2^	Primary	Secondary	χ^2^	Low	High	χ^2^	
Pay attention to physical health										
increased	43.7	52.5	26.24 ***	52.2	47.6	8.38 *	27.3	56.2	28.45 ***	48.4
same as before	49.6	43.3		41.3	47.6		57.1	40.0		46.3
decreased	6.7	4.1		6.5	5.0		15.6	3.8		5.3
2. Pay attention to mental health										
increased	34.2	39.5	10.73 **	37.4	36.8	4.120	18.2	45.2	34.80 ***	36.9
same as before	58.4	54.9		54.4	57.2		62.3	51.0		56.6
decreased	7.5	5.6		8.2	6.0		19.5	3.8		6.5
3. Time spent to rest										
increased	45.9	50.4	5.75	44.3	49.2	11.56 **	40.3	53.0	5.15 ^#^	48.2
same as before	44.6	40.9		43.3	42.5		48.1	40.6		42.7
decreased	9.5	8.7		12.4	8.2		11.7	6.4		9.1
4. Time spent to relax										
increased	49.7	55.5	11.64 **	49.2	53.7	32.03 ***	46.1	60.5	5.26 ^#^	52.7
same as before	42.0	38.5		38.3	40.6		43.4	32.2		40.1
decreased	8.3	6.1		12.4	5.7		10.5	7.3		7.1
5. Time spent to exercise										
increased	24.1	26.0	3.34	30.3	23.6	11.93 **	15.8	31.0	7.21 *	25.0
same as before	41.0	42.3		37.3	42.9		47.4	40.9		41.7
decreased	34.9	31.7		32.4	33.5		36.8	28.1		33.3

^#^ *p* < 0.10, * *p* < 0.05, ** *p* < 0.01, *** *p* < 0.001.

**Table 3:** The positive impact on social support by groups with Chi-Square (χ^2^) Test (*N* = 2863).

%	Gender			Primary or Secondary School			Family Affluence Group			Overall
	Male	Female	χ^2^	Primary	Secondary	χ^2^	Low	High	χ^2^	
Support from friends										
increased	21.0	21.9	7.43 *	24.9	20.5	60.95 ***	10.4	26.7	21.33 ***	21.4
same as before	66.7	69.0		56.7	70.9		66.2	65.7		67.9
decreased	12.3	9.1		18.4	8.6		23.4	7.6		10.6
2. Support from family members										
increased	27.9	27.9	3.22	39.2	24.9	76.96 ***	13.2	34.6	28.93 ***	27.9
same as before	64.0	65.7		49.7	68.9		67.1	61.0		64.9
decreased	8.1	6.4		11.1	6.2		19.7	4.4		7.2
3. Sharing feelings with family members										
increased	22.4	23.5	0.93	26.3	22.2	44.21 ***	9.1	30.8	19.46 ***	23.0
same as before	65.5	65.4		55.5	68.1		68.8	59.4		65.4
decreased	12.0	11.1		18.2	9.8		22.1	9.8		11.5
4. Sharing feelings with others when feeling blue										
increased	17.5	21.9	8.60 *	21.6	19.3	50.17 ***	14.3	26.0	13.82 **	19.8
same as before	66.9	63.8		54.9	68.0		58.4	62.2		65.3
decreased	15.6	14.3		23.5	12.7		27.3	11.7		14.9
5. Caring for family members’ feeling										
increased	35.5	38.9	4.09	46.5	34.8	51.99 ***	27.3	43.5	34.31 ***	37.2
same as before	58.2	55.8		44.5	60.3		53.2	54.0		57.0
decreased	6.3	5.4		9.1	4.9		19.5	2.5		5.8

* *p* < 0.05, ** *p* < 0.01, *** *p* < 0.001.

**Table 4:** The negative impacts on mental health by groups with Chi Square (χ^2^) Test (*N* = 2863).

%	Gender			Primary or Secondary School			Family Affluence Group			Overall
	Male	Female	χ^2^	Primary	Secondary	χ^2^	Low	High	χ^2^	
Being more stressed										
yes	34.0	43.6	29.95 ***	38.5	39.2	0.09	39.0	41.0	0.10	39.1
no	66.0	56.4		61.5	60.8		61.0	59.0		60.9
2. Having larger study pressure										
yes	49.4	66.4	83.31 ***	54.1	59.5	5.63 *	48.1	58.7	2.87 ^#^	58.4
no	50.6	33.6		45.9	40.5		51.9	41.3		41.6
3. Feeling more horrified										
yes	20.0	27.5	22.05 ***	23.1	24.2	0.31	31.2	21.6	3.16 ^#^	24.0
no	80.0	72.5		76.9	75.8		68.8	78.4		76.0
4. Feeling more apprehensive										
yes	26.5	37.1	36.40 ***	28.0	33.2	5.85 *	24.7	30.6	1.04	32.1
no	73.5	62.9		72.0	66.8		75.3	69.4		67.9
5. Feeling more helpless										
yes	18.0	25.1	21.23 ***	20.8	22.0	0.42	20.8	20.1	0.02	21.7
no	82.0	74.9		79.2	78.0		79.2	79.9		78.3

^#^ *p* < 0.10, * *p* < 0.05, *** *p* < 0.001.

**Table 5:** The impacts on perceived vulnerability and safety by groups with independent *t*-test (*N* = 2863).

Mean (s.d)	Gender			Primary or Secondary School			Family Affluence Group			Overall
	Male	Female	*t*-test	Primary	Secondary	*t*-test	Low	High	*t*-test	
Safety in the community	3.85 (1.01)	3.87 (0.90)	−0.37	4.02 (0.98)	3.82 (0.95)	4.53 ***	3.53 (0.82)	3.97 (1.02)	−3.47 **	3.86 (0.96)
Safety at home	4.25 (0.88)	4.27 (0.83)	−0.62	4.38 (0.86)	4.23 (0.85)	3.67 ***	3.96 (0.79)	4.40 (0.84)	−4.15 ***	4.26 (0.85)
Perceived vulnerability	2.11 (0.85)	2.17 (0.75)	−2.10 *	2.18 (0.83)	2.13 (0.79)	1.27	2.07 (0.81)	2.22 (0.80)	−1.52	2.14 (0.80)

* *p* < 0.05, ** *p* < 0.01, *** *p* < 0.001.

**Table 6:** Binary logistic regression on the impact on positive lifestyle changes.

Odd Ratio [95% CI]		More Attention to Physical	More Attention to Mental Health	More Time Spending to Rest	More Time Spending to Relax	More Time Spending to Exercise
Age		0.97 (0.91, 1.03)	1.02 (0.96, 1.09)	1.04 (0.98, 1.11)	1.07 * (1.00, 1.14)	0.96 (0.89, 1.04)
Gender	Female	1.22 * (1.02, 1.46)	1.10 (0.92, 1.32)	1.12 (0.94, 1.33)	1.20 * (1.00, 1.42)	1.15 (0.93, 1.42)
	Male (ref)	1.00	1.00	1.00	1.00	1.00
Family affluence		1.19 *** (1.09, 1.31)	1.29 *** (1.17, 1.42)	1.11 * (1.01, 1.21)	1.12 * (1.03, 1.23)	1.18 ** (1.05, 1.32)
Safety in the community		1.23 *** (1.10, 1.37)	1.19 ** (1.06, 1.33)	1.00 (0.90, 1.11)	1.07 (0.96, 1.19)	1.06 (0.93, 1.20)
Safety at home		1.18 ** (1.04, 1.34)	1.21 ** (1.06, 1.38)	1.19 ** (1.05, 1.35)	1.18 ** (1.04, 1.33)	1.09 (0.94, 1.26)
Perceived vulnerability		1.93 *** (1.70, 2.20)	1.75 *** (1.54, 1.98)	1.23 *** (1.09, 1.39)	1.20 ** (1.07, 1.36)	1.28 *** (1.12, 1.48)
Being more stressed	Yes	1.16 (0.92, 1.45)	1.22 ^#^ (0.97, 1.53)	1.21 ^#^ (0.97, 1.51)	1.40 ** (1.12, 1.75)	0.99 (0.76, 1.30)
	No (ref)	1.00	1.00	1.00	1.00	1.00
Having larger study pressure	Yes	1.17 (0.95, 1.43)	0.85 (0.69, 1.05)	1.16 (0.95, 1.42)	1.04 (0.85, 1.27)	1.04 (0.81, 1.32)
	No (ref)	1.00	1.00	1.00	1.00	1.00
Feeling more horrified	Yes	1.10 (0.83, 1.47)	1.50 ** (1.13, 1.98)	0.92 (0.70, 1.21)	1.03 (0.78, 1.37)	0.89 (0.63, 1.26)
	No (ref)	1.00	1.00	1.00	1.00	1.00
Feeling more apprehensive	Yes	1.38 * (1.07, 1.79)	1.08 (0.83, 1.40)	1.07 (0.82, 1.38)	1.18 (0.91, 1.52)	1.01 (0.74, 1.37)
	No (ref)	1.00	1.00	1.00	1.00	1.00
Feeling more helpless	Yes	0.71 * (0.54, 0.94)	1.08 (0.81, 1.43)	0.84 (0.64, 1.10)	0.79 ^#^ (0.60, 1.04)	1.09 (0.79, 1.52)
	No (ref)	1.00	1.00	1.00	1.00	1.00

^#^ *p* < 0.10, * *p* < 0.05, ** *p* < 0.01, *** *p* < 0.001.

**Table 7:** Binary logistic regression on the positive changes of social support.

Odd Ratio [95% CI]		More Support from Friends	More Support from Family Members	More Sharing of Feelings with Family Members	More Sharing of Feeling with Others when Feeling Blue	More Caring for Family Members’ Feeling
Age		0.87 *** (0.81, 0.94)	0.79 *** (0.74, 0.85)	0.91 * (0.84, 0.98)	0.95 (0.88, 1.03)	0.88 *** (0.82, 0.94)
Gender	Female	0.94 (0.77, 1.15)	1.00 (0.83, 1.22)	0.98 (0.8, 1.20)	1.28 * (1.03, 1.60)	1.05 (0.88, 1.25)
	Male (ref)	1.00	1.00	1.00	1.00	1.00
Family affluence		1.22 *** (1.09, 1.36)	1.20 ** (1.08, 1.33)	1.34 *** (1.20, 1.50)	1.19 ** (1.06, 1.33)	1.11 * (1.01, 1.22)
Safety in the community		1.10 (0.97, 1.25)	1.19 ** (1.05, 1.34)	1.11 (0.98, 1.26)	1.05 * (0.92, 1.21)	1.14 * (1.02, 1.28)
Safety at home		1.20 * (1.03, 1.39)	1.36 *** (1.17, 1.57)	1.15 ^#^ (0.99, 1.34)	1.22 * (1.04, 1.43)	1.37 *** (1.20, 1.57)
Perceived vulnerability		1.30 *** (1.13, 1.49)	1.65 *** (1.45, 1.88)	1.41 *** (1.23, 1.61)	1.4 6*** (1.27, 1.69)	1.53 *** (1.35, 1.73)
Being more stressed	Yes	1.01 (0.78, 1.31)	1.20 (0.94, 1.52)	1.39 * (1.08, 1.78)	1.21 (0.93, 1.59)	1.18 (0.94, 1.47)
	No (ref)	1.00	1.00	1.00	1.00	1.00
Having larger study pressure	Yes	1.02 (0.81, 1.30)	0.92 (0.74, 1.15)	1.02 (0.81, 1.30)	0.83 (0.65, 1.07)	1.16 (0.94, 1.43)
	No (ref)	1.00	1.00	1.00	1.00	1.00
Feeling more horrified	Yes	0.96 (0.69, 1.33)	0.95 (0.70, 1.29)	0.87 (0.64, 1.20)	1.03 (0.73, 1.44)	1.21 (0.91, 1.60)
	No (ref)	1.00	1.00	1.00	1.00	1.00
Feeling more apprehensive	Yes	0.84 (0.62, 1.14)	1.12 (0.85, 1.47)	1.17 (0.88, 1.56)	1.10 (0.80, 1.50)	1.30 * (1.01, 1.68)
	No (ref)	1.00	1.00	1.00	1.00	1.00
Feeling more helpless	Yes	1.65 ** (1.20, 2.26)	0.92 (0.68, 1.24)	0.88 (0.64, 1.21)	1.48 * (1.06, 2.06)	0.82 (0.62, 1.09)
	No (ref)	1.00	1.00	1.00	1.00	1.00

^#^ *p* < 0.10, * *p* < 0.05, ** *p* < 0.01, *** *p* < 0.001.

## Data Availability

The data presented in this study are available on request from the corresponding author. The data are not publicly available due to privacy.
